# *Agaricus bisporus* Crude Extract: Characterization and Analytical Application

**DOI:** 10.3390/molecules25245996

**Published:** 2020-12-18

**Authors:** Maria A. Morosanova, Tatyana V. Fedorova, Alexandra S. Polyakova, Elena I. Morosanova

**Affiliations:** 1 Analytical Chemistry Division, Chemistry Department, Lomonosov Moscow State University, 119234 Moscow, Russia; m.a.morosanova@gmail.com (M.A.M.); conc.lab.student@gmail.com (A.S.P.); 2 Research Center of Biotechnology, A.N. Bach Institute of Biochemistry, Russian Academy of Sciences, 119071 Moscow, Russia; fedorova_tv@mail.ru

**Keywords:** crude *Agaricus bisporus* extract, tyrosinase, two-dimensional electrophoresis, MALDI TOF/TOF MS, phenolic compounds, l-DOPA determination, phenol determination

## Abstract

In the present work crude *Agaricus bisporus* extract (ABE) has been prepared and characterized by its tyrosinase activity, protein composition and substrate specificity. The presence of mushroom tyrosinase (PPO3) in ABE has been confirmed using two-dimensional electrophoresis, followed by MALDI TOF/TOF MS-based analysis. GH27 alpha-glucosidases, GH47 alpha-mannosidases, GH20 hexosaminidases, and alkaline phosphatases have been also detected in ABE. ABE substrate specificity has been studied using 19 phenolic compounds: polyphenols (catechol, gallic, caffeic, chlorogenic, and ferulic acids, quercetin, rutin, dihydroquercetin, l-dihydroxyphenylalanine, resorcinol, propyl gallate) and monophenols (l-tyrosine, phenol, *p*-nitrophenol, *o*-nitrophenol, guaiacol, *o*-cresol, *m*-cresol, *p*-cresol). The comparison of ABE substrate specificity and affinity to the corresponding parameters of purified *A. bisporus* tyrosinase has revealed no major differences. The conditions for spectrophotometric determination have been chosen and the analytical procedures for determination of 1.4 × 10^−4^–1.0 × 10^−3^ M l-tyrosine, 3.1 × 10^−6^–1.0 × 10^−4^ M phenol, 5.4 × 10^−5^–1.0 × 10^−3^ M catechol, 8.5 × 10^−5^–1.0 × 10^−3^ M caffeic acid, 1.5 × 10^−4^–7.5 × 10^−4^ M chlorogenic acid, 6.8 × 10^−5^–1.0 × 10^−3^ M l-DOPA have been proposed. The procedures have been applied for the determination of l-tyrosine in food supplements, l-DOPA in synthetic serum, and phenol in waste water from the food manufacturing plant. Thus, we have demonstrated the possibility of using ABE as a substitute for tyrosinase in such analytical applications, as food supplements, medical and environmental analysis.

## 1. Introduction

Polyphenol oxidases (PPOs) are copper-containing enzymes that are omnipresent among bacteria, fungi, archaea, plants, and animals [1]. The PPO family is composed of three different types of enzymes: catechol oxidases, laccases, and tyrosinases. Catechol oxidases catalyze the oxidation of *o*-diphenols to *o*-quinones (diphenolase activity). Laccases catalyze the oxidation of various aromatic compounds (di- and polyphenols, aminophenols and diamines). Tyrosinases catalyze the ortho-hydroxylation of monophenols (monophenolase activity) and the following diphenolase reaction. The advantages of PPOs, such as the ability to catalyze reactions without additional cofactors and to use dissolved oxygen for the oxidation of phenolic compounds, are widely used for PPO analytical applications [2]. In recent years, the interest in using plant (e.g., banana, sweet potato eggplant, avocado) tissues and crude extracts such as PPO as a source for analytical purposes has been observed [3,4,5]. Plant tissues and crude extracts can be used directly with minimal preparation for both electrochemical and optical biosensors. As a result, the target enzyme (in most plants, catechol oxidase) remains in its natural environment, which increases its stability and, sometimes, its activity, when compared with the corresponding purified enzymes [3]. Other advantages include the simplicity of enzyme handling, which also contributes to the lower cost of the resulting analytical device. Finding cost-effective and efficient biosensors is beneficial not only for the scientific community, but also for society.

Mushroom tissues and crude extracts are less studied than plant extracts for such analytical applications. Mushrooms produce two types of PPOs: laccases (EC 1.10.3.2) and tyrosinases (EC 1.14.18.1). Mushroom laccases are extracellular enzymes. An example of a *Pleurotus ostreatus* crude laccase extract application for catecholamines biosensor construction has been described [6].

Tyrosinase has been found to be widely distributed throughout the phylogenetic tree, including in various mushrooms. Among mushrooms, *Agaricus bisporus* is the most commonly consumed species worldwide, and is also a representative of its family [7]. Its tissue is a rich source of tyrosinase and the high activity extracts of this mushroom can be obtained without extensive purification [8]. *A. bisporus* crude extracts have been extensively used for low-cost biomodification of phenols or bioremediation of phenol-polluted water [8,9,10]. Dephenolization catalyzed by the crude *A. bisporus* extract has been shown to be at least as effective as corresponding purified tyrosinase [9]. The treatment of phenol-containing waters using mushroom tyrosinase extract has been marked by a color change [11], which most likely has been the result of the enzymatic oxidation reaction.

*A. bisporus* tissue has been recently proposed for phenol biosensor construction [12]. Similar to the industrial waters dephenolization, the biosensors containing fresh [13] or lyophilized [14] *A. bisporus* tissue are based on the naturally present mushroom tyrosinase. Thus, *A. bisporus* tissue has been shown to not only be useful for phenol removal, but also for phenol determination.

Mushroom tyrosinase is the most popular among researchers [7]. Purified mushroom tyrosinases (from *A. bisporus* or *Aspergillus niger*) have been employed for optical or electrochemical biosensors for many phenolic compounds determination: l-DOPA [15], sulfamethoxazole [16], acetaminophen [17], tea polyphenols [18], olive oil polyphenols [19], bisphenol A [20]. However, crude *A. bisporus* extract or tissue has not yet been used for the determination of tyrosinase substrates, other than phenol.

Being an available source of high tyrosinase activity, crude *A. bisporus* extract needs to be analyzed in terms of its analogy to tyrosinase in various aspects. Firstly, its protein composition has not been determined yet and the exact enzyme with tyrosinase activity in the extract has not been identified. Secondly, crude *A. bisporus* extract biochemical properties (specificity, affinity) towards various tyrosinase substrates, other than just phenol, have not been studied. The aim of the present work was to prepare crude *A. bisporus* extract, study its protein composition and its substrate specificity and affinity, compare its biochemical properties to purified *A. bisporus* tyrosinase, and, finally, to assess the possibility of using it as a substitute for tyrosinase in such applications as food supplements and in medical and environmental analysis.

## 2. Results and Discussion

### 2.1. Agaricus bisporus Extract Tyrosinase Activity and Protein Composition

The activity of crude *Agaricus bisporus* extract (ABE), prepared in this study, was determined by comparing the reaction speed of catechol oxidation in the presence of ABE and tyrosinase from *Aspergillus niger* (ANT). ANT served as a standard with known activity (3900 U/mg). ABE was characterized by 530 ± 40 U/mL and 1.2 ± 0.3 mg/mL total protein content (*n* = 3, *p* = 0.95). The specific activity of ABE can be calculated as 440 U/mg of protein, while commercial *A. bisporus* tyrosinase (ABT) usually has the specific activity of 1000–8000 U/mg of protein. We can estimate differences in the ABE and ABT activities, using the approach described in [21]: ABT has been shown to have 90 times greater specific activity than the homogenate. ABE had 10 times less specific activity than the average commercial ABT, so, our simple extraction procedure allowed approximately 10-fold purification of the tyrosinase. ABE activity was 3 times higher than for crude eggplant extract that we studied before (880 U/g versus 320 U/g) [5].

Crude extracts are usually more stable than isolated enzymes. ABE kept more than 95% of its activity after 9 months of storage at −20 °C. The first work describing isolation of ABT [22] states that the purification of the enzyme increases its instability. Isolated tyrosinase solutions retained their activity for several days at 4–8 °C and for several weeks at −20 °C [22].

**Figure 1:**
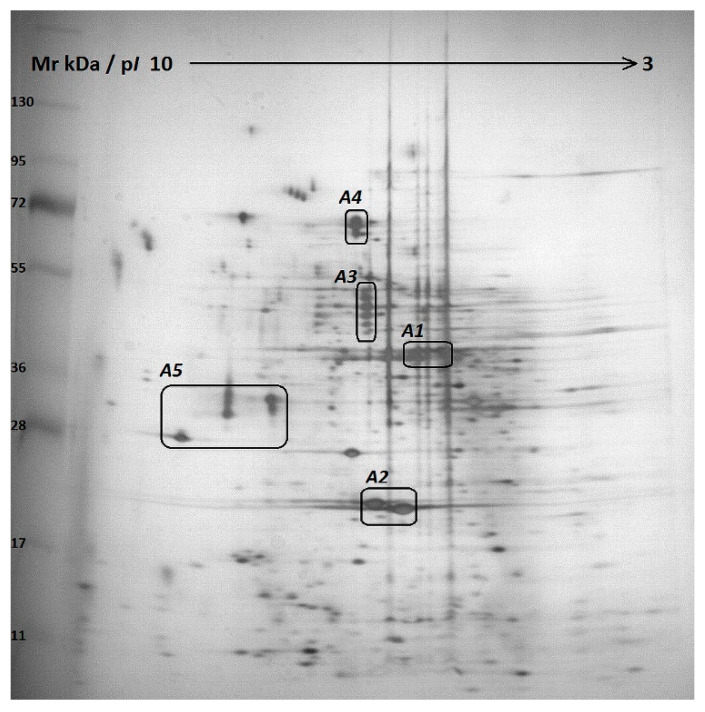
Two dimensional gel electrophoregram of crude *Agaricus bisporus* extract (ABE). The proteins were precipitated from ABE and resolved by 2D electrophoresis. The protein composition of each area (A1–A5) is given in Table 1.

The protein composition of ABE was studied using two-dimensional electrophoresis (2DE) with silver staining (Figure 1). The most abundant proteins in the extract have been identified (protein areas A1–A5 on 2DE gel).

The MALDI TOF/TOF MS based analysis of the tryptic peptides isolated from the area A1 near 40–44 kDa and 5.0–5.5 p*I* on the electrophoregram (Figure 1 A1) resulted in the unambiguous identification of the proteins as different isoforms of polyphenol oxidase 3 (PPO3, tyrosinase) from *A. bisporus* (Table 1 and Table 2, Figure 1). For the first time, PPO3 was cloned, isolated, and characterized in [23]; recently, the crystal structure of the PPO3 tyrosinase was obtained [24].

The amino acid sequences of six polypeptides (PPO1 to 6) are known for a polyphenol oxidase originating from *A. bisporus* [25,26]. It is known that the protein exists as a zymogen, also described as the latent form or pro-tyrosinase with protein mass 68 kDa and an active form with protein mass about 43 kDa [27]. The mature active form is generated by proteolytic cleavage of the latent form, by which the C-terminal part of the protein is removed and the previously buried active site becomes exposed [28,29]. The formation of the active form of the protein by proteolytic cleavage starts immediately after cell lysis, forming great numbers of active tyrosinase species as well as huge amounts of colored compounds (e.g., melanins). Expression studies have shown [26] that different isoforms (PPO1–6) are expressed in different quantities depending on the growth-stage and the fruit-body compartment (e.g., cap, stipe).

In addition to tyrosinase, other proteins have also been identified in ABE, such as AB21 protein (Figure 1 A2), glycoside hydrolase of the 27 and 47 families including GH27 alpha-galactosidase and GH47 alpha-mannosidase (Figure 1 A3), alkaline phosphatase, and GH20 beta-hexosaminidase (Figure 1 A4) as well as hypothetical proteins with unknown functions (Figure 1 A5).

GH27 alpha-galactosidase is a member of the group of exo-glycosidases and is responsible for hydrolysis of galactose-containing oligosaccharide, such as raffinose family oligosaccharides (RFOs). It has diverse applications in the nutraceutical, pharmaceutical industries and other industrial fields such as food and feed, sugar, paper, and pulp for the removal of raffinose and stachyose [30]. They are also important medically for blood group conversion and in the treatment of Fabry disease. Most of the research on α-galactosidases has focused on their isolation from various microbial sources.

GH47 alpha-mannosidases are thought to participate in post-translational modification of secreted proteins [31]. Glycosylation is one of the most ubiquitous posttranslational modifications for eukaryotic proteins. There are numerous examples of the attachment of glycans to carrier proteins resulting in changes in their physicochemical properties, such as solubility or heat stability, as well as physiological properties, such as bioactivity or intra- or intercellular trafficking.

GH20 hexosaminidases are involved in important biological processes catalyzing the hydrolysis of *N*-acetyl-hexosaminyl residues in glycosaminoglycans and glycoconjugates. Such organisms as crustaceans, insects, fungi, and bacteria were shown to have glycoside hydrolases family 20. While in bacteria these enzymes degrade diverse oligosaccharides, providing nitrogen and carbon sources to the catabolism, in the other mentioned organisms, GH20 together with chitinases mainly take part in the degradation of chitin (the most common polysaccharide after cellulose in nature) [32]. In modern technology, GH20 are used as part of biopesticides and biofungicides to prevent harmful actions of insects and fungi, respectively.

Alkaline phosphatase is a phosphomonoesterase enzyme that catalyzes the hydrolysis of monoesters of phosphoric acid (at alkaline pH), yielding phosphate and the corresponding alcohol. The abundance in nature and importance of this enzyme in biological systems has made phosphatase activity assessments one of the most commonly performed enzymatic tests [33]. Alkaline phosphatases are used in enzyme-linked immunoabsorbent assays (ELISA), nonisotopic probing, blotting, and sequencing systems [34].

The recently characterized AB21 protein *A. bisporus* was found in the ABE in significant quantities (Figure 1 A2). The reported crystallographic structure of AB21 protein exhibits a rod-like helical bundle fold with a structural similarity to bacterial toxins of the ClyA superfamily [35]. An abundance of AB21 in the fruiting bodies of *A. bisporus* was demonstrated by immunostaining.

Enzymes of higher fungi have the potential for use in the industry [36]. Some of the enzymes detected in the ABE, such as GH20 hexosaminidase, GH27 alpha-galactosidase and GH47 alpha-mannosidase, have also been recently found in the fruiting bodies of the China edible mushroom *Auricularia polytricha* [37].

ABE protein composition was analyzed for the first time and the presence of tyrosinase enzyme in ABE was confirmed. Other potentially commercially important enzymes were also identified in ABE, which are mostly involved in saccharides biochemical pathways and would not affect phenolic compound oxidation by tyrosinase. Thus, ABE was likely to perform as an excellent substitute for mushroom tyrosinase in various applications.

### 2.2. Agaricus bisporus Extract Substrate Specificity

Intracellular polyphenol oxidases (PPO) can be divided in two groups—tyrosinases that catalyze oxidation of both mono- and polyphenolic compounds and catechol oxidases that only oxidize polyphenolic compounds. Using crude extracts raises the question of the extract selectivity, as well as their comparability to the respective isolated enzymes. To address these issues we proposed comparing the extract properties to the properties of the corresponding isolated enzyme using a range of potential substrates and inhibitors.

Substrate specificity is usually investigated by substrate acceptance assays [1]. In our work, substrate acceptance assays for ABE were performed to check the acceptance of various polyphenolic (catechol, gallic, caffeic, chlorogenic, and ferulic acids, quercetin, rutin, dihydroquercetin, l-dihydroxyphenylalanine (l-DOPA), resorcinol, propyl gallate) and monophenolic compounds (l-tyrosine, phenol, *p*-nitrophenol, *o*-nitrophenol, guaiacol, *o*-cresol, *m*-cresol, *p*-cresol). We investigated ABE activity towards these phenolic compounds spectrophotometrically by recording the spectra of colored oxidation products for 5 to 80 min. Phenolic compounds were considered being oxidized if the differences in spectra were observed with reaction time increase.

**Figure 2:**
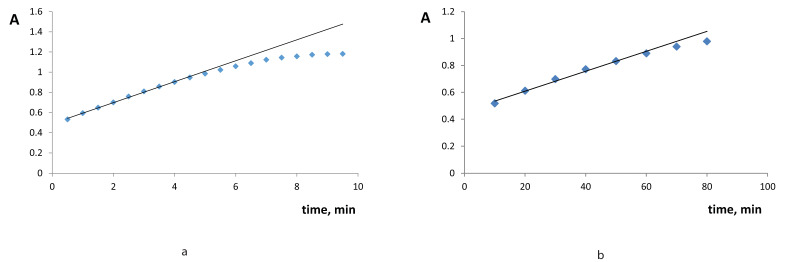
The increase of absorbance with time for 1.0 × 10^−3^ M l-DOPA (**a**) and l-tyrosine (**b**). pH 7.2, ABE activity 265 U (**a**) and 530 U (**b**).

Polyphenolic compounds (catechol, gallic, caffeic, and chlorogenic acids, l-DOPA, cresols, and resorcinol) were rapidly oxidized in the presence of ABE with the formation of yellow colored products (broad absorption 400–450 nm). The catechol oxidase substrate specificity of ABE is similar to the substrate specificity of the plant extracts that we studied before [4,5]: almost all the studied polyphenols were found to be substrates, except well-known PPO and tyrosinase inhibitors like ferulic acid or quercetin. Unlike the previously studied crude eggplant extract, ABE demonstrated significant monophenolase activity towards l-tyrosine and phenol. Red colored products (470 and 500 nm, respectively) were observed starting from 10 min from the reaction start. The identified absorption maxima were used for the further experiments. The reaction for monophenols was slower than for polyphenolic substrates, but still at 60 min the reaction reached equilibrium. The oxidation of both poly- and monophenolic compounds is characteristic of tyrosinase activity and the slower oxidation of monophenolic compounds is generally observed for tyrosinases. Thus, we identified ABE as demonstrating tyrosinase activity rather than catechol oxidase activity, as opposed to the plant extracts that we had studied before. Figure 2 gives the examples of absorbance increase with time for poly- and monophenolic compound oxidation. The oxidation reaction speed (ΔA) was chosen as an analytical signal for further applications. We chose the time intervals where the absorbance was a linear function of the reaction time: 30–270 s for polyphenols and 10–60 min for monophenols.

The results of the ABE substrate acceptance assay (Table 3) were the following: 12 compounds were oxidized (observed by colored products formation) in the presence of ABE (catechol, gallic, caffeic, and chlorogenic acids, dihydroquercetin, l-DOPA, resorcinol, *o*-cresol, *m*-cresol, *p*-cresol, l-tyrosine, phenol) and 7 compounds were not oxidized (ferulic acid, quercetin, rutin, *p*-nitrophenol, *o*-nitrophenol, propyl gallate, guaiacol). We compared ABE substrate specificity to the specificity of corresponding isolated enzyme—tyrosinase (1.14.18.1) from *Agaricus bisporus* (ABT, commercially available tyrosinase). The data on ABT substrate specificity was taken from the BRENDA database [38,39,40,41,42].

We categorized all the studied phenolic compounds in 6 subsets, each represented by a cell in Table 3. For catechol, gallic, caffeic, and ferulic acids, quercetin, rutin, l-DOPA, *p*-cresol, l-tyrosine, and phenol, the results for ABE and ABT were in agreement (the compounds marked as substrates were oxidized in the presence of ABE, and the compounds marked as inhibitors were not oxidized) [38,39,40,41,42]. The gray color in Table 3 represents the two possible contradicting subsets between experimental data from present work and the data from the BRENDA database: 1) compounds oxidized by ABE, but marked as ABT inhibitors; and 2) compounds not oxidized by ABE, but marked as ABT substrates. The first subset was represented by resorcinol and chlorogenic acid, the second subset was empty. Resorcinol was oxidized in the presence of ABE and was named as both substrate and the inhibitor in the database, depending on its concentration [41]. Chlorogenic acid was the only compound that was marked as a tyrosinase inhibitor [42], but was oxidized in the presence of ABE. The possible explanation for this could be based on the differences in the experimental settings. In our work, we studied all the phenolic compound interactions with ABE individually. Data in [42] are obtained by studying the simultaneous interaction of chlorogenic acid and l-DOPA with ABT, and in this case chlorogenic acid behaved as an inhibitor. Chlorogenic acid could still be an ABT substrate, but can perform as a competitive inhibitor for l-DOPA oxidation. Thus, ABE had shown very similar substrate specificity to ABT.

### 2.3. Agaricus bisporus Extract Interaction with Substrates

**Figure 3:**
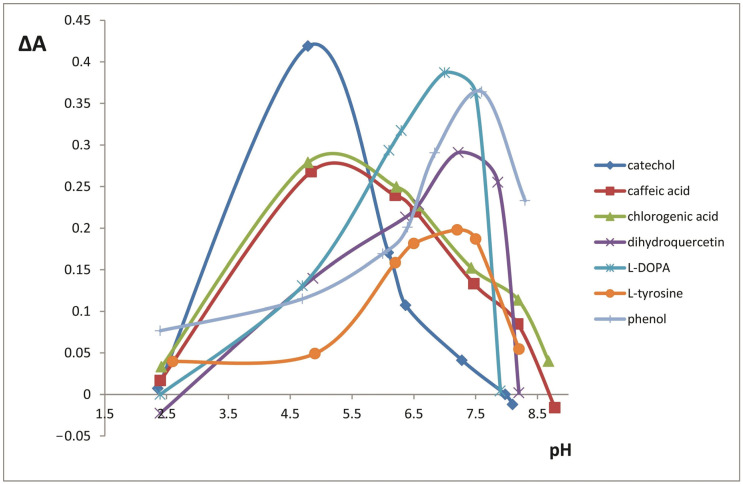
Influence of pH on the oxidation reaction speed (ΔA = A_270 s_ − A_30 s_ for catechol, caffeic acid, chlorogenic acid, dihydroquercetin, and l-DOPA; ΔA = A_60 min_ − A_10 min_ for l-tyrosine and phenol). C(substrate) = 1.0 × 10^−3^ M, ABE activity = 265 U.

**Figure 4:**
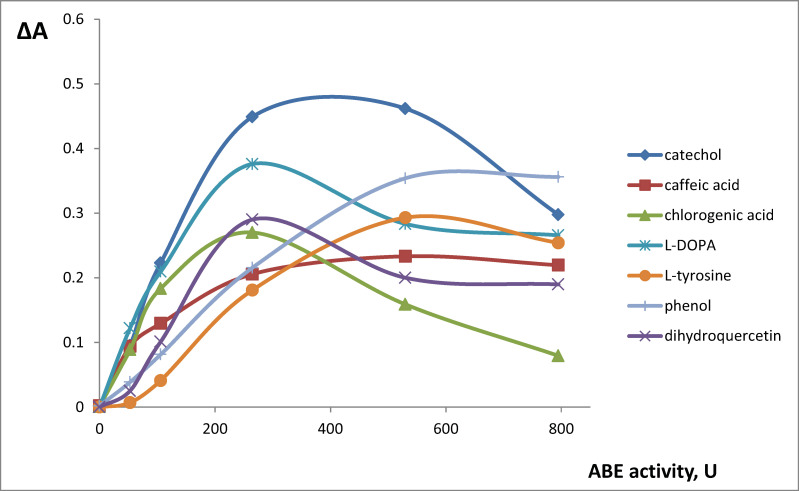
Influence of ABE activity on the oxidation reaction speed (ΔA = A_270 s_ − A_30 s_ for catechol, caffeic acid, chlorogenic acid, dihydroquercetin, and l-DOPA; ΔA = A_60 min_ − A_10 min_ for l-tyrosine and phenol). C (substrate) = 1.0 × 10^−3^ M, pH = 4.8 for catechol, caffeic acid, and chlorogenic acid, pH = 7.2 for dihydroquercetin, l-DOPA, l-tyrosine, and phenol.

In order to choose the reaction conditions, we investigated the influence of pH in the range 2.0–9.0 on the oxidation of phenolic compounds (Figure 3 shows experimental data for selected compounds). The reaction speed reached maximal values in two pH ranges—4.5–5.0 (catechol, caffeic, gallic, and chlorogenic acids, resorcinol, and *o*-cresol) and 7.0–7.5 (dihydroquercetin, l-DOPA, l-tyrosine, phenol, *m*-cresol, and *p*-cresol). In previous studies, ABT was shown to have two pH optima—5.3 and 7.0 [43]. All the further activity measurements were conducted at pH optima for each phenolic compound. Oxidation reaction speed values also depend on the amount of the enzyme (ABE activity) and the time range of the measurement. We investigated this dependence in the time ranges selected for the analytical applications (270 s for polyphenols, 60 min for monophenols) to find the activity that leads to maximal analytical signals (reaction speed). For polyphenolic compounds, the maximal reaction speed was observed for 265 U ABE activity and for monophenolic compounds, the maximal reaction speed was reached at 530 U ABE activity (Figure 4).

**Figure 5:**
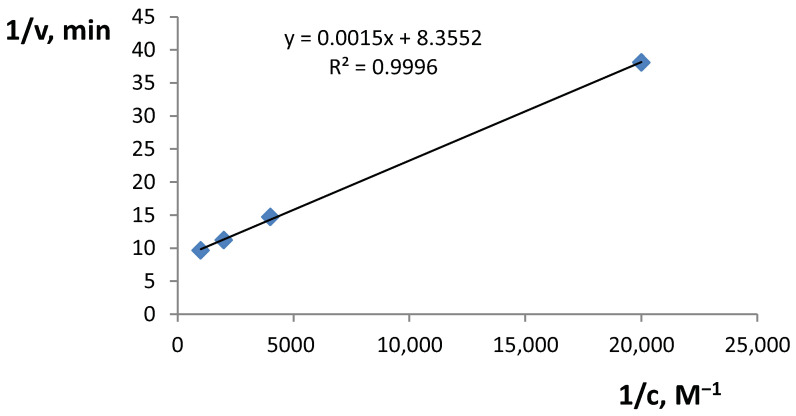
Lineweaver–Burk plot for l-DOPA. pH = 7.2, ABE activity 265 U, reaction speed (v, min^−1^) is (A_270 s_ − A_30 s_)/4.

We compared ABE substrate affinity with ABT substrate affinity. The oxidation reaction speed (min^−1^) was calculated as the absorbance increase in 1 min. Michaelis constants were determined using Lineweaver–Burk plots (the example for l-DOPA is given in Figure 5). In the chosen conditions the Michaelis constants of selected substrates oxidation reactions were determined and compared to those of the purified enzyme (ABT) (Table 4). All the Michaelis constants, except for caffeic acid, were almost identical to the database values, signifying that the oxidation reactions are not largely affected by the presence of non-tyrosinase ABE components.

### 2.4. Analytical Application

The analytical use of crude extracts is a promising approach, as the natural medium provides higher stability of the enzymes and the low cost of crude extracts compared to the purified enzymes allows for a wider application. The elimination of enzyme purification steps reduces chemical consumption and waste generation, consequently leading to more environmentally friendly analytical methods, i.e., “green analytical chemistry” [5]. As we had shown that ABE contained mushroom tyrosinase, a very popular enzyme in analytical chemistry, we decided to propose various possible analytical applications for ABE.

The chosen reaction conditions were used to develop analytical procedures for phenolic compound determination. For monophenolic compounds, the absorbance at 60 min was chosen as the analytical signal, and for faster oxidizing polyphenolic compounds, the difference between absorbance at 30 s and at 270 s after the reaction start (ΔA) was chosen. The analytical ranges (starting from limit of quantitation) and the limit of detection (LOD) values for ABE procedures are given in Table 5.

Analytical procedures were used for selected phenolic compound determination in various samples (Table 6): the examples of food supplements and medical and environmental analytical tasks were chosen. For l-DOPA and phenol, spiked samples were prepared by adding analytes to the corresponding matrix (Table 6, Added). The analytical signal for non-spiked samples did not significantly differ from the blank signal in distilled water (*p* = 0.14 and *p* = 0.06 for synthetic serum and waste water). The results of the determination were compared with the reference values (data given by producer for l-tyrosine and the added amount for l-DOPA and phenol) and good agreement was observed.

The analytical ranges of the developed procedures allowed phenol determination in waste waters, l-tyrosine determination in food supplements, and l-DOPA determination in biological fluids. LOD values are comparable with other spectroscopic procedures using purified tyrosinase [15,44,45,46,47,48] (Table 7). For l-tyrosine, no spectroscopic procedures were found in the literature; however, the comparison with electrochemical determination (LOD 1.0 × 10^−5^ M [49]) allows us to conclude the same. Thus, ABE can successfully substitute purified tyrosinase in such analytical applications.

## 3. Materials and Methods

### 3.1. Chemicals

All chemicals have been purchased from Acros Organics, Morris Plains, NJ, USA and Sigma, St. Louis, MO, USA and were at least of analytical grade. Tyrosinase from *Aspergillus niger* (ANT) (3900 U/mg) was purchased from Sigma, St. Louis, MO, USA (CAS 9002-10-2). Bovine serum albumin (BSA) was purchased from PanEco, Moscow, Russia.

### 3.2. Crude Extract: Preparation and Characterization

Crude extract preparation: in the present work, the procedure for crude *Agaricus bisporus* extract (ABE) preparation was adapted from [4]: ABE was prepared by stirring 150.0 g of homogenized mushroom tissue (purchased from local markets) in 250 mL of 0.07 M phosphate buffer (pH 7.0) at 0 °C for 30 min, then filtering twice through a paper filter.

The activity of the crude extract used in this work has been determined by comparing the reaction speed of catechol oxidation in the presence of ABE and *Aspergillus niger* tyrosinase (ANT) as in [4]. The reaction speed was calculated as 400 nm absorbance change (measured with SPECTROstar Nano spectrophotometer (BMG Labtech, Ortenberg, Germany)). We conducted all the experiments at room temperature (22 °C).

ABE 1.0 mL aliquots were stored at −20 °C. ABE activity remained unchanged after at least 6 months of storage.

Total protein measurements were performed by the Biuret method as in [4]. Bovine serum albumin stock solution (2.5 mg/mL) was prepared with doubly distilled water. The calibration curve was prepared by mixing 2.0 mL of BSA solution in the 0.1–2.5 mg/mL concentration range with 1.0 mL of Biuret reagent; blank was prepared by mixing 2.0 mL of doubly distilled water with 1.0 mL of Biuret reagent. The absorbance was measured at 540 nm using SPECTROstar Nano spectrophotometer (BMG Labtech, Ortenberg, Germany). The total protein content in ABE was determined in triplicate.

Isolation of proteins: Extract sample was filtered through syringe filters (0.45 μm, Sartorius, Goettingen, Germany) and then concentrated 10-fold and simultaneously desalted by tangential flow ultrafiltration system Pellicon^®^ XL with a 5 kDa MW cut-off membrane Biomax 5 (Millipore, Burlington, MA, USA). The proteins were then precipitated using the Acetone-TCA method, which included protein precipitation with acetone containing 13.3% trichloroacetic acid (TCA) and 0.093% β-mercaptoethanol (at 1:1 sample/precipitant ratio) for 12 h at −20 °C. The precipitate was separated by centrifugation (15 min, 4400 rpm), washed twice by 0.07% β-mercaptoethanol in acetone and dried for 5 min in airflow at 20 °C.

The proteins were dissolved in a resolubilization solution containing 5% Servalytes (pH 3–10; Serva Electrophoresis, Heidelberg, Germany), 1% 1,4-dithiothreitol (DTT), 4% 3-[(3-cholamidopropyl) dimethyl-ammonio]-1-propanesulfonate (CHAPS), 7 M urea, 2 M thiourea and treated in an ultrasonic bath (Bandelin Sonorex, 35 kHz, 120 W) for 10 min at 20 °C. After the treatment, the reaction mixture was incubated for 1 h at 20 °C and centrifuged for 5 min at 9000 g. The supernatant was collected and used for further studies.

Two-dimensional electrophoresis: 2D electrophoresis was performed as described in [50] using a Protean II xi 2-D Cell system (Bio-Rad, Hercules, CA, USA). During the isoelectrofocusing (IEF), the pH gradient varied from 3 to 10 (Servalytes; Serva Electrophoresis, Heidelberg, Germany). The sample amount was 150–200 μg protein per tube. The IEF was performed for 16 h under the following regimens: 100 V for 45 min, 200 V for 45 min, 300 V for 45 min, 400 V for 45 min, 500 V for 45 min, 600 V for 45 min, 700 V for 10 h, 900 V for 1.5 h.

The subsequent electrophoresis of samples resulting upon the IEF was performed in a gradient (7.5–25%) polyacrylamide gel in the presence of SDS at 300 V. After IEF, the samples were incubated for 15 min in solution containing 6 M urea, 2% SDS, 10 mM DTT, 0.5 M Tris-HCl (pH 6.8) to prevent oxidation of sulfhydryl groups of the proteins. To visualize the distribution of protein components, the gels were stained with silver nitrate and Brilliant Blue R Staining Solution (Sigma, St. Louis, MO, USA) for mass spectrometry.

For protein mapping the Infinity1000/26MX gel-documenting system (Vilber Lourmat, Collégien, France) was used. The protein maps were analyzed using the ImageMaster 2D Platinum v.7 program (GE Healthcare, Danderyd, Sweden).

MALDI TOF/TOF MS analysis: To perform mass spectrometry, gel pieces 3–4 mm^3^ in size were cut out and washed twice to remove the dye in 100 μL of 40% solution of acetonitrile in 0.1 M ammonium hydrocarbonate for 20 min at 37 °C. Upon removal of the solution, 100 μL of acetonitrile was added for dehydrating the gel. After removal of acetonitrile and drying the gel, a 15 μg/mL solution of modified trypsin (Promega, Madison, WI, USA) in 0.05 M ammonium hydrocarbonate was added. The sample was hydrolyzed for 8 h at 37 °C, then 0.5% trifluoroacetic acid (TFA) in 10% solution of aqueous acetonitrile was added. The protein hydrolysate containing solution above the gel was used for mass spectrometry; 2,5-dihydroxybenzoic acid (Aldrich, St. Louis, MO, USA) solution (10 mg/mL) in 20% aqueous acetonitrile with 0.5% TFA was used as matrix.

Mass spectra were obtained using a Bruker Ultraflex II MALDI TOF/TOF mass spectrometer (Bremen, Germany) equipped with a UV laser (Nd) in positive ion regimen with a reflectron; the accuracy of measured monoisotopic masses upon recalibration by trypsin autolysis peaks was 0.005% (50 ppm). The spectra were obtained in the mass range of 7004500 *m*/*z* at a laser power chosen for optimal resolution. The fragmentation spectra were obtained using a tandem regimen of the device, and the accuracy on measurement of fragmented ions was no less than 1 Da.

Mass spectra were processed using the FlexAnalysis 3.3 program (Bruker Daltonics, Bremen, Germany). Proteins were identified with the Mascot program (www.matrixscience.com), and using the peptide mass fingerprinting the search was carried out in the NCBI database (www.ncbi.nlm.nih.gov) among proteins of all organisms at the abovementioned accuracy and taking into account possible oxidation of methionine residues by air oxygen and possible modification of cysteine residues in the polyacrylamide gel. Candidate proteins with score >83 in the NCBI database were considered reliably determined (*p* < 0.05), whereas proteins with score >50 were considered probable. The search for homologous proteins was performed with the Biotools 3.2 program (Bruker Daltonics, Bremen, Germany) by combined MS + MS/MS results.

Additionally, sequences of the peptides individually derived from the fragmentation data were analyzed by BLAST NCBI using the fungal subset of the GenBank database.

### 3.3. General Procedure for Crude Agaricus bisporus Extract and Phenolic Compounds Interaction Study

The following polyphenolic (catechol, gallic, caffeic, chlorogenic, and ferulic acids, quercetin, rutin, dihydroquercetin, l-dihydroxyphenylalanine (l-DOPA), resorcinol, propyl gallate) and monophenolic compounds (l-tyrosine, phenol, *p*-nitrophenol, *o*-nitrophenol, guaiacol, *o*-cresol, *m*-cresol, *p*-cresol) were studied. Stock solutions of phenolic compounds (1.0 × 10^−2^ M) were prepared with doubly distilled water. Only freshly prepared solutions of phenolic compounds were used.

Phosphate, oxalate, phthalate, and borate buffer solutions were used. pH of the reaction mixtures was measured with a HI83303 photometer/pH-meter and an HI11310 pH electrode (Hanna Instruments, Woonsocket, RI, USA).

The influence of the reaction conditions were studied: 2.0 mL of phenolic compound solution (1.0 × 10^−6^–1.0 × 10^−2^ M) was mixed with 0.5 mL of buffer solution (pH 2.0–9.0), 0.1–1.5 mL ABE (prepared as described in “Crude extract preparation”), and the spectra of the colored reaction products were recorded for 4 min for polyphenols and for 60 min for monophenols, the total volume of the probe remained 4.0 mL. Spectra of colored products of enzymatic oxidation of phenolic compounds were recorded with SPECTROstar Nano spectrophotometer (BMG Labtech, Ortenberg, Germany). Spectra were analyzed with MARS software (BMG Labtech, Ortenberg, Germany).

Michaelis constants (K_m_) were calculated using Lineweaver–Burk plots.

### 3.4. Analytical Application: Calibration Curves

In all the applications, ABE prepared as described in “Crude extract preparation” was used.

In order to obtain the calibration curves, we determined the dependence of the analytical signal (absorbance for slower monophenolic compounds oxidation and reaction speed for faster polyphenolic compounds oxidation) on the analyte concentration in the 1.0 × 10^−5^–1.0 × 10^−3^ M range for catechol, caffeic and chlorogenic acids, l-DOPA and l-tyrosine, and in the 1.0 × 10^−6^–1.0 × 10^−4^ M range for phenol. The limit of detection (LOD) was calculated as 3·standard deviation of the blank absorbance (*n* = 3) divided by the slope value. The limit of quantitation (LOQ) was calculated as 3·LOD and served as the lower limit of the analytical range. The upper limit was chosen so the calibrations were linear (R ≥ 0.99).

The procedures for each phenolic compound are given below:

Catechol, caffeic, and chlorogenic acids determination: 2.0 mL of standard solution was mixed with 0.5 mL of phthalate buffer (pH 4.0), then 0.5 mL of ABE was added and the absorbance was measured after 30 s and 270 s at 400 nm using SPECTROstar Nano spectrophotometer (BMG Labtech, Ortenberg, Germany) and the difference of the absorbance values was used as an analytical signal.

l-DOPA determination: 2.0 mL of standard solution was mixed with 0.5 mL of phosphate buffer (pH 8.0), then 0.5 mL of ABE was added and the absorbance was measured after 30 s and 270 s at 470 nm using SPECTROstar Nano spectrophotometer (BMG Labtech, Ortenberg, Germany) and the difference of the absorbance values was used as an analytical signal.

l-tyrosine determination: 2.0 mL of standard solution was mixed with 0.5 mL of phosphate buffer (pH 8.0), then 1.5 mL of ABE was added and the absorbance was measured after 60 min at 470 nm using SPECTROstar Nano spectrophotometer (BMG Labtech, Ortenberg, Germany).

Phenol determination: 7.0 mL of standard solution was mixed with 0.5 mL of phosphate buffer (pH 8.0), then 0.2 mL of ABE was added and the absorbance was measured after 60 min at 500 nm (l 2.0 cm) using a KFK-3 spectrophotometer (ZOMZ, Sergiev Posad, Russia).

### 3.5. Analytical Application: Samples Analysis

Treated waste water was obtained from the food manufacturing plant in the Moscow region. Phenol spiked samples were prepared by dissolving phenol in the waste water.

l-tyrosine capsule (Twinlab Corporation, Boca Raton, FL, USA) content was dissolved in the doubly distilled water.

Synthetic serum was prepared as in [51]. l-DOPA spiked samples were prepared by dissolving l-DOPA in the synthetic serum.

Phenol determination: 7.0 mL of sample was mixed with 0.5 mL of phosphate buffer (pH 8.0), then 0.2 mL of ABE was added and the absorbance was measured after 60 min at 500 nm (*l* 2.0 cm) using a KFK-3 spectrophotometer (ZOMZ, Sergiev Posad, Russia). Phenol in treated waste water samples was determined in triplicate using a calibration curve in the 3.0 × 10^−6^ to 1.0 × 10^−4^ M range.

l-tyrosine determination: 2.0 mL of sample was mixed with 0.5 mL of phosphate buffer (pH 8.0), then 1.5 mL of ABE was added and the absorbance was measured after 60 min at 470 nm using a SPECTROstar Nano spectrophotometer (BMG Labtech, Ortenberg, Germany). l-tyrosine in food supplement sample was determined in triplicate using a calibration curve in the 1.0 × 10^−4^ to 1.0 × 10^−3^ M range.

l-DOPA determination: 2.0 mL of sample was mixed with 0.5 mL of phosphate buffer (pH 8.0), then 0.5 mL of ABE was added and the absorbance was measured after 30 s and 270 s at 470 nm using a SPECTROstar Nano spectrophotometer (BMG Labtech, Ortenberg, Germany). The difference of the absorbance values was used as an analytical signal. l-DOPA in synthetic serum samples was determined in triplicate using a calibration curve in the 5.0 × 10^−5^ to 2.5 × 10^−4^ M range.

## 4. Conclusions

In the present work, we have characterized crude *Agaricus bisporus* extract (ABE) tyrosinase activity, protein composition and substrate specificity. ABE has demonstrated three times greater activity than previously studied crude plant extracts (eggplant, banana). ABE protein composition has been studied using two-dimensional electrophoresis (2DE) with silver staining, followed by MALDI TOF/TOF MS-based analysis. The presence of mushroom tyrosinase (PPO3) in ABE has been confirmed. Our assay has also detected other proteins in ABE, including GH27 alpha-glucosidases, GH47 alpha-mannosidases, GH20 hexosaminidases, and alkaline phosphatases, offering other possible industrial applications of ABE in the future.

The possibility of using ABE as a source of tyrosinase for analytical applications has been studied. We have investigated ABE tyrosinase-like substrate specificity by performing substrate acceptance assays for 20 phenolic compounds, and the obtained data are in good agreement with BRENDA database data for purified *A. bisporus* tyrosinase. ABE has kept more than 95% of its activity after 9 months of storage at −20 °C. Substrate affinity (Michaelis constants) has also been determined for ABE catalyzed oxidation and they have been similar to purified *A. bisporus* tyrosinase-related constants. For further ABE evaluation of tyrosinase as a source for analytical applications, we have proposed spectrophotometric procedures for several important analytical tasks (the determination of phenol in waste waters, l-tyrosine in food supplements, and l-DOPA in biological fluids). The obtained LOD values have been found to be comparable to the LOD values observed for purified tyrosinase-based spectroscopic analytical procedures. Thus, we have concluded that crude *Agaricus bisporus* extract can be used in various analytical applications as an available and stable substitute for tyrosinase. Such cost-effective and reliable biosensing systems are not only valuable as the scientific results, but also have a high societal impact.

## Figures and Tables

**Table 1:** Proteins identified by MALDI TOF/TOF mass spectrometry in *Agaricus bisporus* crude extract (ABE).

Identified Proteins	Accession Number	Highest Similarity to Proteins	Score (−10lgP)
Protein identification data of area A1 on the electrophoregram			
Polyphenol oxidase ^a,b^ (Tyrosinase)	2Y9W_A	Chain A, Crystal Structure of PPO3, A Tyrosinase from *Agaricus bisporus*	344
	C7FF04	Polyphenol oxidase 3 (Tyrosinase) *Agaricus bisporus*; PPO3; Precursor	297
	ADE67053	Tyrosinase (*Agaricus bisporus*)	282
Protein identification data of area A2 on the electrophoregram			
Protein AB21 ^a,b^	XP_006461442 ^c^	hypothetical protein AGABI2DRAFT_192945 (*Agaricus bisporus var. bisporus* H97)	335
Protein identification data of area A3 on the electrophoregram			
Alpha-galactosidase (GH27) ^a,b^ Alpha-mannosidases (GH47) ^a,b^	XP_006456345.1 ^d^ XP_007331943.1 ^e^	Mixture 1: Components: 1. hypothetical protein AGABI2DRAFT_228269 (*Agaricus bisporus var. bisporus* H97); 2. hypothetical protein AGABI1DRAFT_86588 (*Agaricus bisporus var. burnettii* JB137-S8)	240
Alpha-galactosidase (GH27) ^a,b^ Alpha-mannosidases (GH47) ^a,b^	XP_007334630.1 ^f^ XP_007331943.1 ^e^	Mixture 2: Components: 1. hypothetical protein AGABI1DRAFT_80761 (*Agaricus bisporus var. burnettii* JB137-S8); 2. hypothetical protein AGABI1DRAFT_86588 (*Agaricus bisporus var. burnettii* JB137-S8)	221
Alpha-galactosidase (GH27) ^a,b^ Alpha-mannosidases (GH47) ^a,b^	XP_006456345.1 ^d^ XP_007331943.1 ^e^	Mixture 1: Components: 1. hypothetical protein AGABI2DRAFT_228269 (*Agaricus bisporus var. bisporus* H97); 2. hypothetical protein AGABI1DRAFT_86588 (*Agaricus bisporus var. burnettii* JB137-S8)	246
Alpha-galactosidase (GH27) ^a,b^ Mannosyl-oligosaccharide alpha-1,2-mannosidase 1B (GH47) ^a,b^	XP_006456345.1 ^d^ XP_006463333.1 ^g^	Mixture 2: Components: 1. hypothetical protein AGABI2DRAFT_228269 (*Agaricus bisporus var. bisporus* H97); 2. hypothetical protein AGABI2DRAFT_194179 (*Agaricus bisporus var. bisporus* H97)	230
Protein identification data of area A4 on the electrophoregram			
Alkaline phosphatase ^a,b^	XP_006462861.1 ^h^	hypothetical protein AGABI2DRAFT_72252 (*Agaricus bisporus var. bisporus* H97)	242
Beta-hexosaminidase (GH20) ^a,b^	XP_006462439.1 ^i^	hypothetical protein AGABI2DRAFT_193587 (*Agaricus bisporus var. bisporus* H97)	193
Molecular chaperone DnaK ^a,b^	WP_031376016.1	MULTISPECIES: molecular chaperone DnaK (Pantoea)	463
Protein identification data of area A5 on the electrophoregram			
Hypothetical protein ^a,b^	XP_007329073.1	hypothetical protein AGABI1DRAFT_113037 (*Agaricus bisporus var. burnettii* JB137-S8)	319
Hypothetical protein ^a,b^	XP_006456974.1	hypothetical protein AGABI2DRAFT_195909 (*Agaricus bisporus var. bisporus* H97)	187
Hypothetical protein ^a,b^	XP_006463886.1	hypothetical protein AGABI2DRAFT_194569 (*Agaricus bisporus var. bisporus* H97)	465
Thaumatin-like protein ^a,b^	XP_006455282.1 ^j^	hypothetical protein AGABI2DRAFT_226703 (*Agaricus bisporus var. bisporus* H97)	188

^a^ Mascot Peptide Mass Fingerprint. ^b^ BLAST analysis of MSMS-derived peptide sequences. ^c^ Similar sequence was annotated as toxin-like protein (*Agaricus bisporus var. bisporus*) AWL54261.1. ^d^ Similar sequence was annotated as glycoside hydrolase Family 27 protein, alpha-galactosidase precursor (*Leucoagaricus gongylophorus*) CDJ79834.1. ^e^ Similar sequence was annotated as glycoside hydrolase Family 47 protein, alpha-mannosidases (*Termitomyces sp.* J132) KNZ78347.1. ^f^ Similar sequence was annotated as glycoside hydrolase Family 27 protein, putative alpha-galactosidase B (*Leucoagaricus sp.* SymC.cos) KXN84638.1. ^g^ Similar sequence was annotated as glycoside hydrolase Family 47 protein, putative mannosyl-oligosaccharide alpha-1,2-mannosidase 1B (*Leucoagaricus sp.* SymC.cos) KXN84060.1. ^h^ Similar sequence was annotated as alkaline phosphatase protein (*Moniliophthora roreri* MCA 2997) ESK93059.1. ^i^ Similar sequence was annotated as glycoside hydrolase Family 20 protein (*Laccaria bicolor* S238N-H82) XP_001876141.1; beta-hexosaminidase 2 (*Leucoagaricus sp*. SymC.cos) KXN85407.1. ^j^ Similar sequence was annotated as thaumatin-like protein (*Armillaria gallica*) PBL03081.1.

**Table 2:** List of peptides matches found by MALDI TOF/TOF MS measurements (protein spots tryptic digest of the Area 1 on the electrophoregram, Figure 1).

	Sequence	Mr (expt)	Mr (calc)	*m*/*z* (Observed)	ppm
1	R.ALQVLQAR.D	897.5477	897.5396	898.5549	9.01
2	R.EWTFNMLTK.N	1168.5607	1168.5587	1169.5680	1.73
3	K.NRLNILDFVK.N	1230.6811	1230.7084	1231.6884	−22.18
4	K.SVYINDWVHK.H	1259.6255	1259.6299	1260.6328	−3.44
5	K.NDKFFTLYVR.A	1301.6726	1301.6768	1302.6799	−3.26
6	K.SLMPLVGIPGEIK.N	1352.7572	1352.7737	1353.7645	−12.24
7	R.FTTSDQAEWIQAAK.D	1594.7632	1594.7627	1595.7705	0.31
8	K.SLMPLVGIPGEIKNR.L	1622.9171	1622.9178	1623.9243	−0.44
9	K.AAAPGFREWTFNMLTK.N	1838.9170	1838.9138	1839.9243	1.78
10	R.FTTSDQAEWIQAAKDLR.Q	1978.9743	1978.9748	1979.9816	−0.28
11	R.YPDVQKQENIEGMIAGIK.A	2032.0332	2032.0299	2033.0405	1.64
12	R.LNILDFVKNDKFFTLYVR.A	2244.2282	2244.2307	2245.2355	−1.10
13	R.AYESTWEQTLWEAAGTVAQR.F	2296.0962	2296.0760	2297.1035	8.79
14	R.LLALWQTMNYDVYVSEGMNR.E	2402.1470	2402.139	2403.1543	2.99
15	K.FHPIEPTFEGDFAQWQTTMR.Y	2437.1231	2437.1161	2438.1303	2.84
16	K.QVEITDYNGTKIEVENPILHYK.F	2602.2997	2602.3279	2603.3070	−10.82
17	R.EATMGLIPGQVLTEDSPLEPFYTK.N	2635.3001	2635.3091	2636.3074	−3.42
18	R.DQSDYSSFFQLGGIHGLPYTEWAK.A	2745.2632	2745.2711	2746.2704	−2.89
19	R.DKQVEITDYNGTKIEVENPILHYK.F	2845.4231	2845.4498	2846.4303	−9.38
20	R.QPFWDWGYWPNDPDFIGLPDQVIR.D	2960.3728	2960.3922	2961.3801	−6.58
21	K.FHPIEPTFEGDFAQWQTTMRYPDVQK.Q	3167.4399	3167.4811	3168.4472	−13.00
22	K.NQDPWQSDDLEDWETLGFSYPDFDPVK.G	3242.3693	3242.3993	3243.3766	−9.25
23	K.DLRQPFWDWGYWPNDPDFIGLPDQVIR.D	3344.5434	3344.6044	3345.5507	−18.22
24	K.NQDPWQSDDLEDWETLGFSYPDFDPVKGK.S	3427.4427	3427.5157	3428.4500	−21.30
25	K.DLRQPFWDWGYWPNDPDFIGLPDQVIRDK.Q	3587.6262	3587.7263	3588.6335	−27.89
26	K.NQDPWQSDDLEDWETLGFSYPDFDPVKGKSK.E	3642.7296	3642.6427	3643.7369	23.9
27	R.DPTLDPLVPGHMGSVPHAAFDPIFWMHHCNVDR.L	3778.6609	3778.7596	3779.6682	−26.11
28	K.NYTWELFSNHGAVVGAHANSLEMVHNTVHFLIGR.D	3819.7564	3819.8692	3820.7637	−29.53
29	K.FHPIEPTFEGDFAQWQTTMRYPDVQKQENIEGMIAGIK.A	4450.9378	4451.1355	4451.9451	−44.41

**Table 3:** Substrate specificity (ABE—crude *Agaricus bisporus* extract, ABT isolated tyrosinase from *Agaricus bisporus*). Gray color represents the two possible contradicting subsets between experimental data from the present work and the data from the BRENDA database: (1) compounds oxidized by ABE, but marked as ABT inhibitors; and (2) compounds not oxidized by ABE, but marked as ABT substrates.

	Experimental Data (Present Work)	Oxidized in the Presence of ABE	Not Oxidized in the Presence of ABE
Data from BRENDA Database			
ABT substrate		Catechol Gallic acid Caffeic acid l-DOPA Resorcinol *p*-Cresol l-tyrosine Phenol	
ABT inhibitor		Chlorogenic acid Resorcinol	Ferulic acid Quercetin Rutin
No data for ABT		Dihydroquercetin *o*-Cresol *m*-Cresol	*p*-Nitrophenol *o*-Nitrophenol Propyl gallate Guaiacol

**Table 4:** Michaelis constants (K_m_) for crude *Agaricus bisporus* extract (ABE) and isolated tyrosinase from *Agaricus bisporus* (ABT).

Phenolic Compound	Km, M	
	ABE	ABT [38]
Catechol	2.7 × 10^−4^	2.5 × 10^−4^
Caffeic acid	2.3 × 10^−4^	1.7 × 10^−3^
Chlorogenic acid	2.1 × 10^−4^	-
l-DOPA	1.8 × 10^−4^	1.7 × 10^−4^
l-tyrosine	7.2 × 10^−4^	2.0 × 10^−4^
Phenol	1.5 × 10^−4^	3.0 × 10^−4^

**Table 5:** Analytical parameters of phenolic compounds determination procedures using crude *A. bisporus* extract (*n* = 3 for LOD).

Phenolic Compound	Analytical Signal	LOD, M	Analytical Range, M
l-tyrosine	Absorbance at 60 min	4.7 × 10^−5^	1.4 × 10^−4^–1.0 × 10^−3^
Phenol		1.0 × 10^−6^	3.1 × 10^−6^–1.0 × 10^−4^
Catechol	The difference of the absorbance values at 30 s and 270 s	1.8 × 10^−5^	5.4 × 10^−5^–1.0 × 10^−3^
Caffeic acid		2.8 × 10^−5^	8.5 × 10^−5^–1.0 × 10^−3^
Chlorogenic acid		4.9 × 10^−5^	1.5 × 10^−4^–7.5 × 10^−4^
l-DOPA		2.3 × 10^−5^	6.8 × 10^−5^–1.0 × 10^−3^

**Table 6:** Phenolic compounds determination (*n* = 3, *p* = 0.95).

Analyte	Sample	Found (RSD, %)	Added
Phenol	Spiked waste water 1	(2.8 ± 0.3) × 10^−6^ M (4.7)	2.9 × 10^−6^ M
	Spiked waste water 2	(7.8 ± 0.7) × 10^−6^ M (3.4)	7.1 × 10^−6^ M
	Spiked waste water 3	(1.4 ± 0.2) × 10^−5^ M (4.4)	1.4 × 10^−5^ M
l-tyrosine	Food supplement	633 ± 147 mg/capsule (9.3)	500 mg/capsule *
l-DOPA	Spiked synthetic serum 1	(7.1 ± 2.3) × 10^−5^ M (23.7)	5.0 × 10^−5^ M
	Spiked synthetic serum 2	(2.5 ± 0.3) × 10^−4^ M (7.9)	2.5 × 10^−4^ M

* producer data.

**Table 7:** Comparison of spectroscopic tyrosinase-based procedures for phenol and l-DOPA determination.

Analyte	Enzyme	Analytical Signal	LOD, M	Reference
Phenol	Mushroom tyrosinase immobilized in chitosan film	Absorbance of the colored products of the enzymatic oxidation followed by coupling with MBTH	1.0 × 10^−6^	[44]
	Mushroom tyrosinase immobilized in PVA matrix	Fluorescence of Ru salts that can be quenched by the oxygen participating in the enzymatic oxidation	8.0 × 10^−5^	[45]
	Mushroom tyrosinase immobilized in graphene oxide film	Surface plasmon resonance angle shift caused by the products of the enzymatic oxidation	1.0 × 10^−6^	[46]
	Mushroom tyrosinase immobilized in hydrogel	CdSe/ZnS quantum dots fluorescence quenching by the product of the enzymatic oxidation (quinone)	1.0 × 10^−6^	[47]
	*Agaricus bisporus* crude extract	Absorbance of the colored products of the enzymatic oxidation	1.0 × 10^−6^	Present work
l-DOPA	*Agaricus bisporus* tyrosinase immobilized in the polyelectrolyte layers	Absorbance of the colored products of the enzymatic oxidation	2.3 × 10^−5^	[15]
	*Amorphophallus companulatus* tyrosinase immobilized in agarose film	Reflectance of the colored products of the enzymatic oxidation	1.7 × 10^−5^	[48]
	*Agaricus bisporus* crude extract	Absorbance of the colored products of the enzymatic oxidation	2.3 × 10^−5^	Present work

## References

[1] Kampatsikas I., Bijelic A., Rompel A. (2019). Biochemical and structural characterization of tomato polyphenol oxidases provide novel insights into their substrate specificity. Sci. Rep..

[2] Gul I., Ahmad M.S., Naqvi S.M.S., Hussain A., Wali R., Farooqi A.A., Ahmed I. (2017). Polyphenol oxidase (PPO) based biosensors for detection of phenolic compounds: A Review. J. Appl. Biol. Biotechnol..

[3] Fatibello-Filho O., Lupetti K.O., Leite O.D., Vieira I.C. (2007). Electrochemical biosensors based on vegetable tissues and crude extracts for environmental, food and pharmaceutical analysis. Compr. Anal. Chem..

[4] Morosanova M.A., Fedorov A.S., Morosanova E.I. (2019). Crude Plant Extracts Mediated Polyphenol Oxidation Reactions in the Presence of 3-Methyl-2-Benzothiazolinone Hydrazone for the Determination of Total Polyphenol Content in Beverages. Curr. Anal. Chem..

[5] Morosanova M.A., Bashkatova A.S., Morosanova E.I. (2019). Spectrophotometric and Smartphone-Assisted Determination of Phenolic Compounds Using Crude Eggplant Extract. Molecules.

[6] Leite O.D., Fatibello-Filho O., Barbosa A.d.M. (2003). Determination of catecholamines in pharmaceutical formulations using a biosensor modified with a crude extract of fungi laccase (*Pleurotus ostreatus*). J. Braz. Chem. Soc..

[7] Seo S.Y., Sharma V.K., Sharma N. (2003). Mushroom tyrosinase: Recent prospects. J. Agric. Food Chem..

[8] Kameda E., Langone M.A.P., Coelho M.A.Z. (2006). Tyrosinase extract from *Agaricus bisporus* mushroom and its *in natura* tissue for specific phenol removal. Environ. Technol..

[9] Atlow S.C., Bonadonna-Aparo L., Klibanov A.M. (1984). Dephenolization of industrial wastewaters catalyzed by polyphenol oxidase. Biotechnol. Bioeng..

[10] Duran N., Esposito E. (2000). Potential applications of oxidative enzymes and phenoloxidase-like compounds in wastewater and soil treatment: A review. Appl. Catal. B.

[11] Ikehata K., Nicell J.A. (2000). Color and toxicity removal following tyrosinase-catalyzed oxidation of phenols. Biotechnol. Prog..

[12] Silva L.M.C., Salgado A.M., Coelho M.A.Z. (2010). *Agaricus bisporus* as a source of tyrosinase for phenol detection for future biosensor development. Environ. Technol..

[13] Silva L.M.C., Salgado A.M., Coelho M.A.Z. (2011). Development of an amperometric biosensor for phenol detection. Environ. Technol..

[14] Silva L.M.C., de Mello A.C.C., Salgado A.M. (2014). Phenol determination by an amperometric biosensor based on lyophilized mushroom (*Agaricus bisporus*) tissue. Environ. Technol..

[15] Fiorentino D., Gallone A., Fiocco D., Palazzo G., Mallardi A. (2010). Mushroom tyrosinase in polyelectrolyte multilayers as an optical biosensor for *o*-diphenols. Biosens. Bioelectron..

[16] del Torno-de Roman L., Alonso-Lomillo M.A., Dominguez-Renedo O., Arcos-Martinez M.J. (2016). Tyrosinase based biosensor for the electrochemical determination of sulfamethoxazole. Sens. Actuators B.

[17] Frangu A., Pravcova K., Silarova P., Arbneshi T., Sys M. (2019). Flow injection tyrosinase biosensor for direct determination of acetaminophen in human urine. Anal. Bioanal. Chem..

[18] Cortina-Puig M., Munoz-Berbel X., Calas-Blanchard C., Marty J.L. (2010). Diazonium-functionalized tyrosinase-based biosensor for the detection of tea polyphenols. Microchim. Acta..

[19] Nadifiyine S., Haddam M., Mandli J., Chadel S., Blanchard C.C., Marty J.L., Amine A. (2013). Amperometric biosensor based on tyrosinase immobilized on to a carbon black paste electrode for phenol determination in olive oil. Anal. Lett..

[20] Wu L., Deng D., Jin J., Lu X., Chen J. (2012). Nanographene-based tyrosinase biosensor for rapid detection of bisphenol A. Biosens. Bioelectron..

[21] Wichers H.J., Gerritsen Y.A.M., Chapelon C.G.J. (1996). Tyrosinase isoforms from the fruitbodies of *Agaricus bisporus*. Phytochemistry.

[22] Kertesz D., Zito R. (1965). Mushroom polyphenol oxidase I. Purification and general properties. Biochim. Biophys. Acta..

[23] Wu J., Chen H., Gao J., Liu X., Cheng W., Ma X. (2010). Cloning, characterization and expression of two new polyphenol oxidase cDNAs from *Agaricus. bisporus*. Biotechnol. Lett..

[24] Ismaya W.T., Rozeboom H.J., Weijn A., Mes J.J., Fusetti F., Wichers H.J., Dijkstra B.W. (2011). Crystal structure of *Agaricus bisporus* mushroom tyrosinase: Identity of the tetramer subunits and interaction with tropolone. Biochemistry.

[25] O’Connor E., McGowan J., McCarthy C.G.P., Amini A., Grogan H., Fitzpatrick D.A. (2019). Whole Genome Sequence of the Commercially Relevant Mushroom Strain *Agaricus bisporus var. bisporus* ARP23. G3-Genes Genom. Genet..

[26] Weijn A., Bastiaan-Net S., Wichers H.J., Mes J.J. (2013). Melanin biosynthesis pathway in *Agaricus bisporus* mushrooms. Fungal Genet. Biol..

[27] Mauracher S.G., Molitor C., Michael C., Kragl M., Rizzi A., Rompel A. (2014). High level protein-purification allows the unambiguous polypeptide determination of latent isoform PPO4 of mushroom tyrosinase. Phytochemistry.

[28] Faccio G., Arvas M., Thony-Meyer L., Saloheimo M. (2013). Experimental and bioinformatic investigation of the proteolytic degradation of the C-terminal domain of a fungal tyrosinase. J. Inorg. Biochem..

[29] Fujieda N., Murata M., Yabuta S., Ikeda T., Shimokawa C., Nakamura Y., Hata Y., Itoh S. (2012). Multifunctions of MelB, a fungal tyrosinase from *Aspergillus oryzae*. Chem. Bio. Chem..

[30] Katrolia P., Rajashekhara E., Yan Q., Jiang Z. (2014). Biotechnological potential of microbial α-galactosidases. Crit. Rev. Biotechnol..

[31] Mast S.W., Moremen K.W. (2006). Family 47 α-Mannosidases in *N*-Glycan Processing. Methods Enzymol..

[32] Val-Cid C., Biarnes X., Faijes M., Planas A. (2015). Structural-Functional Analysis Reveals a Specific Domain Organization in Family GH20 Hexosaminidases. PLoS ONE.

[33] Rankin S.A., Christiansen A., Lee W., Banavara D.S., Lopez-Hernandez A. (2010). Invited review: The application of alkaline phosphatase assays for the validation of milk product pasteurization. J. Dairy Sci..

[34] Miggiano G.A., Mordente A., Martorana G.E., Meucci E., Castelli A. (1983). In vitro effect of ascorbic acid on bovine kidney alkaline phosphatase activity. Acta. Vitaminol. Enzymol..

[35] Komarek J., Kavkova E.I., Houser J., Horackova A., Zdanska J., Demo G., Wimmerova M. (2018). Structure and properties of AB21, a novel *Agaricus bisporus* protein with structural relation to bacterial pore-forming toxins. Proteins.

[36] Erjavec J., Kos J., Ravnikar M., Dreo T., Sabotic J. (2012). Proteins of higher fungi—from forest to application. Trends Biotechnol..

[37] Jia D., Wang B., Li X., Peng W., Zhou J., Tan H., Tang J., Huang Z., Tan W., Gan B. (2017). Proteomic Analysis Revealed the Fruiting-Body Protein Profile of *Auricularia polytricha*. Curr. Microbiol..

[38] Selinheimo E., Gasparetti C., Mattinen M., Steffensen C., Buchert J., Kruus K. (2009). Comparison of substrate specificity of tyrosinases from *Trichoderma reesei* and *Agaricus bisporus*. Enzyme Microb. Technol..

[39] Munoz-Munoz J.L., Garcia-Molina F., Garcia-Ruiz P.A., Molina-Alarcon M., Tudela J., Garcia-Canovas F., Rodriguez-Lopez J.N. (2008). Phenolic substrates and suicide inactivation of tyrosinase: Kinetics and mechanism. Biochem. J..

[40] Karioti A., Protopappa A., Megoulas N., Skaltsa H. (2007). Identification of tyrosinase inhibitors from *Marrubium velutinum* and *Marrubium cylleneum*. Bioorg. Med. Chem..

[41] Garcia-Jimenez A., Teruel-Puche J.A., Berna J., Rodriguez-Lopez J.N., Tudela J., Garcia-Ruiz P.A., Garcia-Canovas F. (2016). Characterization of the action of tyrosinase on resorcinols. Bioorg. Med. Chem..

[42] Selinheimo E., NiEidhin D., Steffensen C., Nielsen J., Lomascolo A., Halaouli S., Record E., OBeirne D., Buchert J., Kruus K. (2007). Comparison of the characteristics of fungal and plant tyrosinases. J. Biotechnol..

[43] Gouzi H., Benmansour A. (2007). Partial purification and characterization of polyphenol oxidase extracted from *Agaricus bisporus* (JE Lange) Imbach. IJCRE.

[44] Abdullah J., Ahmad M., Karuppiah N., Heng L.Y., Sidek H. (2006). Immobilization of tyrosinase in chitosan film for an optical detection of phenol. Sens. Actuators B.

[45] Wu X.J., Choi M.M., Wu X.M. (2004). An organic-phase optical phenol biosensor coupling enzymatic oxidation with chemical reduction. Analyst.

[46] Hashim H.S., Fen Y.W., Omar N.A.S., Daniyal W.M.E.M.M., Saleviter S., Abdullah J. (2020). Structural, optical and potential sensing properties of tyrosinase immobilized graphene oxide thin film on gold surface. Optik.

[47] Jang E., Son K.J., Kim B., Koh W.G. (2010). Phenol biosensor based on hydrogel microarrays entrapping tyrosinase and quantum dots. Analyst.

[48] Paranjpe P., Dutta S., Karve M., Padhye S., Narayanaswamy R. (2001). A disposable optrode using immobilized tyrosinase films. Anal. Biochem..

[49] Rivas G.A., Solis V.M. (1991). Indirect electrochemical determination of l-tyrosine using mushroom tyrosinase in solution. Anal. Chem..

[50] Vasina D.V., Pavlov A.R., Koroleva O.V. (2016). Extracellular proteins of *Trametes hirsuta* st. 072 induced by copper ions and a lignocellulose substrate. BMC Microbiol..

[51] Uppuluri P., Dinakaran H., Thomas D.P., Chaturvedi A.K., Lopez-Ribot J.L. (2009). Characteristics of Candida albicans Biofilms Grown in a Synthetic Urine Medium. J. Clin. Microbiol..

